# Intracranial non-germinomatous germ cell tumors in children and adolescents: how can the experience from an uppermiddle-income country contribute to the worldwide effort to improve outcomes?

**DOI:** 10.3389/fonc.2024.1308128

**Published:** 2024-03-04

**Authors:** Andrea M. Cappellano, Natalia Dassi, Bruna M. Mançano, Sidnei Epelman, Daniela B. Almeida, Sergio Cavalheiro, Patricia A. Dastoli, Maria T. S. Alves, Jardel M. Nicacio, Marcos D. S. Costa, Frederico A. Silva, Simone S. Aguiar, Maria L. Figueiredo, Michael Chen, Nasjla S. Silva, Jonathan L. Finlay

**Affiliations:** ^1^ Pediatric Oncology, Pediatric Oncology Institute-GRAACC/Federal University of São Paulo, São Paulo, Brazil; ^2^ Pediatric Oncology, Hospital do Amor Barretos, Barretos, Brazil; ^3^ Pediatric Oncology, Hospital Santa Marcelina-TUCCA, São Paulo, Brazil; ^4^ Nursing Department, Pediatric Oncology Institute-GRAACC, Federal University of São Paulo, São Paulo, Brazil; ^5^ Neurosurgery Department, Pediatric Oncology Institute-GRAACC, Federal University of São Paulo, São Paulo, Brazil; ^6^ Pathology Department, Pediatric Oncology Institute-GRAACC, Federal University of São Paulo, São Paulo, Brazil; ^7^ Radiology Department, Pediatric Oncology Institute-GRAACC, Federal University of São Paulo, São Paulo, Brazil; ^8^ Radiotherapy Department, Pediatric Oncology Institute-GRAACC, Federal University of São Paulo, São Paulo, Brazil; ^9^ Paediatric Oncology, The Ohio State University, Columbus, OH, United States

**Keywords:** intracranial germ cell tumors (iGCTs), middle income countries (MIC), reduced radiotherapy, autologous stem cell transplantation (ASCT), non-germinomatous cell tumor

## Abstract

**Background:**

Non-germinomatous germ cell tumors (NGGCT) accounts for one third of intracranial GCT. While the germinoma group have an excellent overall survival, the standard of practice for children with NGGCT is still under evaluation.

**Aims:**

Describe the results of the of the Brazilian consortium protocol.

**Methods:**

Since 2013, 15 patients with a diagnosis of NGGCT by histopathology and/or serum/cerebrospinal fluid (CSF) tumor markers, βHCG >200mlU/ml and/or positive alpha-fetoprotein were treated with neoadjuvant chemotherapy with carboplatin, cyclophosphamide and etoposide followed by ventricular radiotherapy (RTV) of 18Gy with boost (32Gy) to the primary site. Metastatic patients underwent craniospinal irradiation (CSI) and “slow responders” to the four initial cycles of CT, to autologous stem cell transplantation (ASCT) followed by CSI.

**Results:**

Mean age, 13.1 years. Thirteen males. Primary sites: pineal (n=12), suprasellar (n=2) and bifocal (n=1). Four patients were metastatic at diagnosis. Eight patients had CSF and/or serum alpha-fetoprotein levels > 1,000ng/ml. Tumor responses after chemotherapy demonstrated complete in six cases and partial in seven, with “second-look” surgery being performed in five cases, and two patients presenting viable lesions being referred to ASCT. The main toxicity observed was hematological grades 3/4. Two patients with metastatic disease, one with Down Syndrome and AFP > 1,000ng/ml and the other with choriocarcinoma and pulmonary metastases, developed progressive disease resulting in death, as well as two other patients without evidence of disease, due to endocrinological disorders. Event-free and overall survival at 2 and 5 years were 80% and 72.7%, respectively, with a mean follow-up of 48 months (range, 7-107).

**Conclusions:**

Despite the small number of patients, in our series, treatment with six cycles of chemotherapy and RTV with focal boost for localized disease (n=11) and ACST for identified slow responders (n=2) seem to be effective strategies contributing to the overall effort to improve outcomes of this group of patients.

## Introduction

Non-germinomatous germ cell tumors (NGGCT) account for one-third of intracranial germ cell tumors (GCT) and encompass various subtypes, including embryonal carcinoma, endodermal sinus tumor, choriocarcinoma, teratoma and mixed tumors (1, 2). Patients in the germinoma group experience excellent overall survival, ongoing research is focused on evaluating treatment strategies to reduce late-effects through less intensive regimens (3). However, the standard of care for children, adolescents and young adults with NGGCT is still under evaluation in order to enhance outcomes.

The objective of this study is to describe a cohort of patients uniformly diagnosed and treated within an upper-middle-income country (UMIC) with chemotherapy and reduced-dose and volume radiotherapy (RTV) followed by autologous stem cell transplantation (ASCT) as a front-line strategy in a subset of patients considered as “slow responders” after initial induction chemotherapy.

## Patients and methods

A prospective trial, conducted by a Brazilian consortium, enrolled patients diagnosed with primary intracranial germ cell tumors and treated at the IOP/GRAACC/Federal University of São Paulo (UNIFESP), Hospital Amor de Barretos, and Hospital Santa Marcelina/TUCCA between 2013 and 2021. Data collection and analysis were performed in December 2022. The germinoma stratum was recently published in *JCO Global Oncology* (4).

The primary and secondary objectives of this study were to determine the event-free survival (EFS) and overall survival (OS) at 2 and 5 years of follow-up from diagnosis for patients with intracranial NGGCT; to assess the impact on survival by reducing the RT dose and volume in the proposed treatment group; to examine the impact of ACST on the survival of NGGCT patients identified as “slow responders”; and to implement “second-look” surgery for patients who did not achieve complete radiological response and observe its impact on overall survival.

The diagnosis of NGGCT and staging included cranial and spinal magnetic resonance imaging (MRI) as well as lumbar cerebrospinal fluid (CSF) cytology and tumor marker assessment at baseline, unless clinically contraindicated. In cases where AFP was detectably elevated (usually serum [5-10 ng/dL] or CSF [2-5 ng/dL]) or there was a significant CSF elevation of βHCG exceeding 200 IU/L, NGGCT was considered diagnostic without the need for histological confirmation. However, all patients with negative tumor markers underwent tumor biopsy for histopathological diagnosis. The chemotherapy plan consisted of an outpatient platinum-based regimen administered in six cycles every 21 days. The cycles were as follows: two consecutive days of carboplatin (300mg/m2 on Days 1 and 2) and etoposide (225mg/m2 on Days 1 and 2), alternating with two consecutive days of cyclophosphamide (1200mg/m2 on Days 1 and 2) and etoposide (225mg/m2 on Days 1 and 2). The cycles containing cyclophosphamide required the use of mesna (sodium 2-mercaptoethane sulfonate) and 5-hour hyperhydration at 1500ml/m2 each day. Assessment of disease was conducted after the completion of every two cycles, involving the monitoring of serum and CSF tumor markers and radiological evaluation through craniospinal MRI.

Patients who showed no evidence of progressive disease during the six cycles of induction chemotherapy proceeded to receive adjusted radiotherapy (RTV) as described below. However, if a residual lesion persisted after the fourth cycle of chemotherapy, a second surgical resection was strongly recommended. In cases where residual non-germinomatous (NG) disease was detected and/or positive tumor markers persisted, the recommended course of action was referral for ACST) followed by CSI. For patients with localized disease, radiotherapy comprised whole ventricular field irradiation (WVFI) at a dose of 18 Gy, with an additional 32 Gy administered as a boost to the primary site. Patients diagnosed with metastatic disease received CSI totaling 36 Gy, with a 20 Gy boost to the primary site. Following ACST irradiation included 30 Gy for the craniospinal region, with a 20 Gy boost to the primary site.

Tumor measurements and responses were assessed according to the revised RECIST criteria (Response Evaluation Criteria in Solid Tumors) (5): Complete Response (CR) was defined as no radiological evidence of tumor and normalization of both serum and lumbar CSF tumor markers; Partial Response (PR) as a 50% reduction in the product of the two greatest tumor diameters on imaging and a reduction of previously elevated tumor marker levels in both serum and lumbar CSF; Minor Response (MR) as a 25-50% reduction in imaging and some reduction of previously elevated serum and lumbar CSF tumor markers; Stable disease (SD) as less than a 25% decrease in imaging size, and Progressive disease (PD) as a 25% increase in tumor size or increasing elevations of either βHCG or AFP in either serum or lumbar CSF.

The Common Terminology Criteria for Adverse Events Version 4.0 was used to categorize adverse events.

Event-free survival (EFS) was defined as the duration from the time of study enrollment to disease progression, disease relapse, the occurrence of a second neoplasm, or death from any cause. Overall survival (OS) was defined as the interval from diagnosis to death due to any cause or the last follow-up visit. Nonparametric curves were generated using the product-limit (Kaplan-Meier) estimator, and these calculations were performed using IBM SPSS software for Windows (version 29.0).

## Results

### Patient characteristics

A total of 58 patients were enrolled in the study, with 15 diagnosed with NGGCT. The median age at diagnosis was 13.1 years, with a range from 5.9 to 16.1 years. Of these patients, 13 (86.6%) were male.

The primary tumor sites were distributed as follows: 12 patients had pineal tumors, two had suprasellar tumors and one had a bifocal tumor. In addition to positive tumor markers, 10 patients received a histopathological diagnosis. Eight patients had AFP levels exceeding 1,000 ng/ml in their CSF and/or serum. Four patients presented with disseminated disease, with three cases located in the ventricular area and one in the thalamus along with extra-CNS involvement in the pulmonary region. Please refer to **Table 1** for a detailed presentation of patient characteristics.

**Table 1:** Patient’s characteristic.

Cases	Age (Y)	Sex	Primary site	Metastasis	Tumor Markers		Pathology
#1	12.6	F	Bifocal	Periventricular	AFP+*	βHCG +**	Germinoma
#2	6.3	F	Suprasellar	No	AFP+	βHCG +	–
#3	13.7	M	Suprasellar	Periventricular	AFP-	βHCG +**	Choricarcinoma
#4	14.1	M	Pineal	No	AFP+*	βHCG +**	Endodermal sinus
#5	8.5	M	Pineal	No	AFP+*	βHCG +**	Germinoma
#6	15.5	M	Pineal	No	AFP+*	βHCG +**	–
#7	10.7	M	Pineal	No	AFP+*	βHCG +**	Choriocarcinoma
#8	14	M	Pineal	No	AFP+	βHCG +**	Germinoma
#9	8	M	Pineal	Periventricular	AFP+*	βHCG +**	Endodemal sinus
#10	5.9	M	Pineal	No	AFP+	βHCG +	–
#11	8.6	M	Pineal	Thalamus/Lung	AFP-	βHCG +**	Choricarcinoma+Embryonal Ca
#12	15.5	M	Pineal	No	AFP+	βHCG +**	–
#13	10.5	M	Pineal	No	AFP+*	βHCG +**	Geminoma
#14	16.1	M	Pineal	No	AFP+*	βHCG +	–
#15	13.3	M	Pineal	No	AFP-	βHCG +**	Geminoma

*AFP> 1000UIml/L **βHCG >200mlU/ml.

### Treatment outcomes and toxicities

All patients successfully completed the scheduled induction chemotherapy cycles every 21 days and received radiation therapy (RT) according to the proposed protocol. The mean interval between end of chemotherapy and initial RT was 30 days (range, 15-90 days). The patient with the longest interval developed febrile neutropenia and septicemia with prolonged intensive care hospitalization. After the completion of induction chemotherapy, six patients achieved complete responses (three after two cycles and three after four cycles), while seven patients achieved partial responses (PR). Unfortunately, two patients experienced disease progression (PD), one during induction, the other following CSI.

Among the patients who achieved partial responses (PR), five underwent “second-look” surgery. The surgical findings included one case with teratoma, one with both choriocarcinoma and germinoma components, and three with fibrosis without any signs of viable tumor in the sampled tissues. The remaining two patients who achieved PR following induction chemotherapy exhibited negative tumor markers and had minimal unresectable residual lesions.

**Figure 1:**
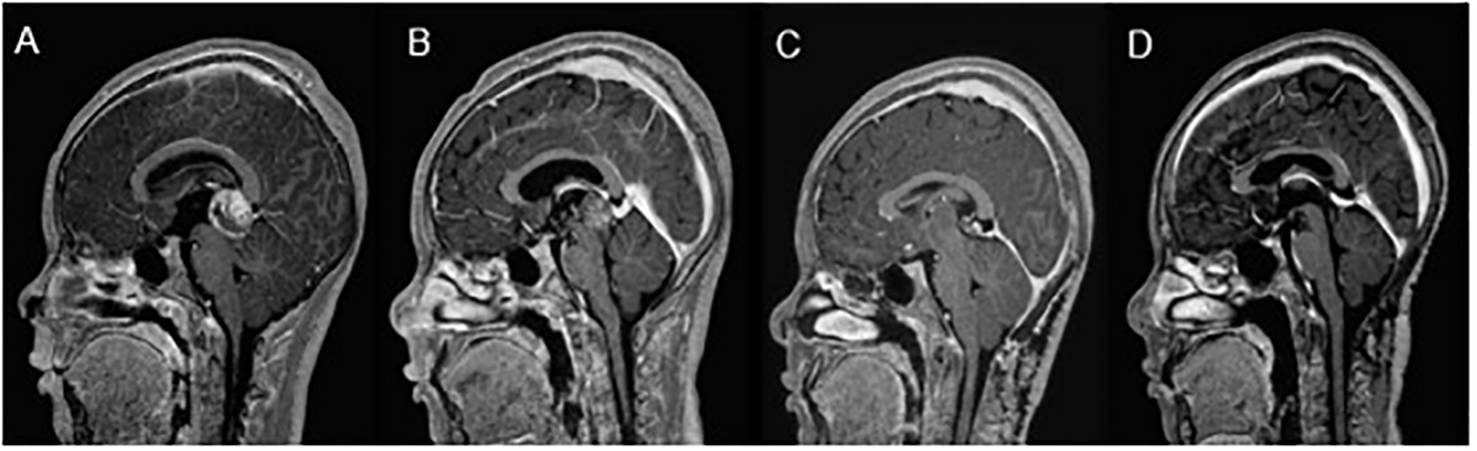
NGGCT “slow responder”. Patient’s journey from diagnosis (CSF βHCG β-10382 mIU/ml; serum-4912 mlU/ml); Biopsy - Germinoma **(A)** to partial response (CSF βHCG 810 mlU/ml) after 2 and 4 cycles of treatment **(B)**, followed by further reduction post second-look surgery (CSF βHCG β- 84 mlU/ml serum-undetectable); Biopsy - Germinoma, **(C)**. Complete response post-autologous stem cell transplantation, disease-free for 8.9 years **(D)**.

Two patients underwent ASCT after completing four cycles of chemotherapy, due to persistently elevated tumor markers and the presence of residual non-germinomatous (NG) components (see **Figure 1**). Both patients initially presented with elevated CSF AFP levels and/or serum levels exceeding 1,000 ng/ml at the time of diagnosis. They are currently alive without disease or recurrence, with event-free survival (EFS) durations of 77 and 107 months, respectively.

**Figure 2:**
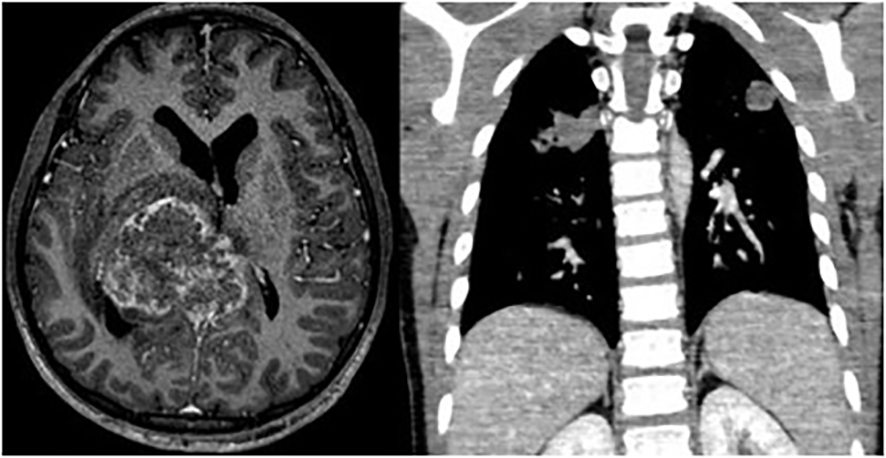
Pineal tumor with thalamic and pulmonary metastasis (βHCG> 10.000UI/ml).

Among patients with progressive disease, one had metastatic tumor involvement in the thalamus and lungs, elevated CSF/serum βHCG (>10,000 IUm/L), and a biopsy confirming choriocarcinoma and embryonal carcinoma elements. This patient experienced spontaneous primary tumor bleeding, resulting in neurological deterioration, and progressed despite chemotherapy, ultimately passing away after seven months during the induction chemotherapy (see **Figure 2**). The other patient, who had Down Syndrome and ventricular dissemination, presented with elevated CSF and serum AFP levels (>10,000 ng/mL). Although achieving a partial response (PR) after chemotherapy with an inoperable scar (negative tumor markers), this patient experienced disease progression locally with positive tumor markers, one month after CSI radiotherapy. Despite receiving additional cancer-directed therapies, he progressed after two cycles of Ifosfamide, carboplatin and etoposide and then two cycles of GEMPOX (6) and passed away 20 months after the initial diagnosis.

Two patients experienced non-disease-related deaths due to electrolyte disturbances attributed to sodium imbalances secondary to diabetes insipidus, after completion of all tumor-directed therapy. EFS for the two patients were nine and 25 months.

**Figure 3:**
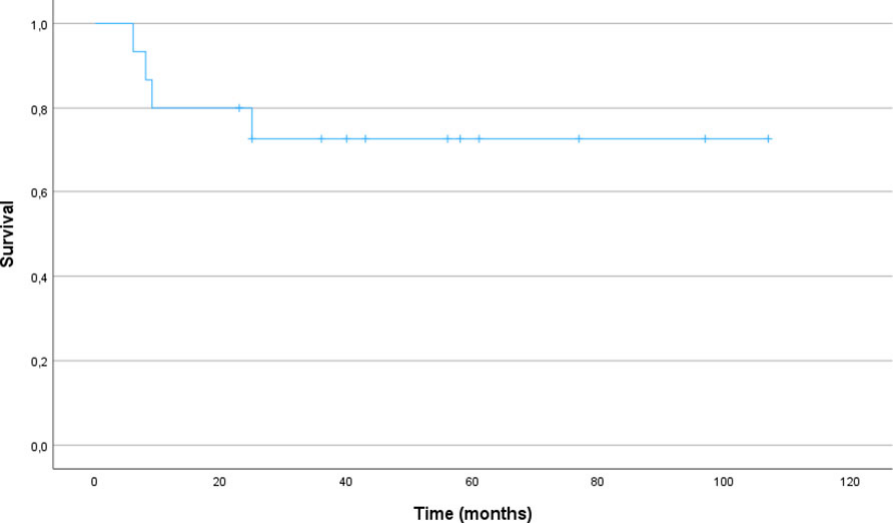
Event-free and Overall Survival (n=15).

Among the eight patients with AFP levels exceeding 1,000 ng/ml in the CSF and/or serum, only one, a patient with Down syndrome, succumbed to disease progression as described above. The other patient deaths were unrelated to disease progression. In the entire cohort, the event-free survival (EFS) and overall survival (OS) rates at 2 and 5 years are 80% and 72.7%, respectively, with a median follow-up of 48 months (range: 7-107) (see **Figure 3**).

The most prevalent toxicities observed were grade 3/4 hematologic toxicities, primarily anemia (n = 15), neutropenia (n = 49) and thrombocytopenia (n = 34) across all assessable cycles. Febrile neutropenia occurred in twelve episodes, with three cases involving documented bloodstream infections. Additionally, three patients experienced electrolyte disturbances, resulting in two toxicity-related deaths.

## Discussion

This study represents the largest prospective trial involving intracranial NGGCT in an upper-middle-income country (UMIC). Our aim is to provide insights into a cohort of patients who received uniform treatment, addressing the challenges posed by social and cancer care disparities while contributing to global efforts to enhance outcomes for this rare group of intracranial tumors.

NGGCT typically occurs in male children during middle school years and predominantly manifests in the pineal region (1, 2), consistent with our series. Bifocal tumors are infrequent in the NG group, and in our series, we identified only one patient with bifocal disease and positive tumor markers. Nevertheless, this underscores the importance of biopsy in such cases, particularly when tumor markers are negative (7, 8).

Primary intracranial germinomas have an excellent overall survival, with the standard approach involving neoadjuvant chemotherapy and reduced-dose whole-ventricular field radiotherapy to minimize late effects without compromising outcomes (9–11). In contrast, historically, NGGCT patients have had a poor prognosis (2). Both radiotherapy-only and chemotherapy-only strategies (12–14) have been deemed inadequate, with platinum-containing combinations as neoadjuvant chemotherapy followed by radiotherapy recognized as an effective treatment (15–17).

Nevertheless, the standard radiotherapy protocol after induction chemotherapy remains a subject of debate. For patients with complete response (CR) and localized disease, several cooperative groups are investigating the ideal radiotherapy field (18–20). For instance, SIOP-96 (18) reported a five-year progression-free survival of 72% for localized tumors (n=116) with local radiotherapy. The Children’s Oncology Group (COG) ACNS0122 (19) employed craniospinal irradiation (CSI) of 36Gy and 54Gy boost, achieving a five-year event-free survival (EFS) of 92% (n=102), while subsequent study ACNS1123 (20) used whole-ventricular radiotherapy (WVRT) with 30.6Gy and 23.4Gy focal boost, reducing the CSI irradiation for localized disease, resulting in an 89% two-year EFS (n=107). Notably, the pattern of treatment failure varied among these studies, with local failures being more common in SIOP and ACNS0122, and spinal cord failures occurring in COG ACNS1123. However, a significant pooled analysis suggests that focal/WVI radiotherapy is not associated with an increased risk of metastatic relapse (21).

Despite a small patient cohort, in our series, 18Gy WVRT with a boost for local disease (n=11) yielded positive responses with no relapses over a five-year period. CSI was reserved for metastatic patients. Regarding dose reduction of WVRT, it appears to be feasible as only a few relapses of NGGCT occur in the ventricles after focal RT, and most of them are attributed to the germinoma component. Breen et al. (22) reported three patients with ventricular relapses, all with a germinoma component, and Murray et al. (23) reported three out of five patients with a germinoma component and one with βHCG between 50-200 IU/L, which would be considered a diagnosis of germinoma in our series. In a separate initiative from the recently initiated ACNS2021, which includes whole ventricular and spinal canal irradiation (WVSCI) for all patients, the plan for our next Brazilian protocol will be to assess alternative treatment intensification strategies while retaining WVRT for patients with localized tumors.

While some patients achieved CR after induction chemotherapy, a subset required “second-look” surgery and treatment intensification. Recognizing these “slow responders” is crucial for prognosis. The SIOP-96 trial revealed worse survival for NGGCT patients with end-of-treatment residual disease, even after “second-look” surgery (18). COG ACNS0122 documented two patients undergoing ASCT (19), a strategy supported by subsequent ACNS1123 (20) considering the great number of patients who did not attain complete responses after induction chemotherapy.

The benefit of this approach as part of the initial treatment for NGGCT remains unclear, due to the small number of patients described, necessitating collaborative efforts and consortia to encompass a larger, uniformly diagnosed, and treated patient population. In our series, seven patients exhibited partial responses after four cycles of chemotherapy, with two showing residual viable tumors and positive tumor markers, qualifying them as “slow responders.” They underwent ASCT and achieved favorable outcomes, with disease-free survival of 77 and 107 months.

Another established prognostic factor is a serum and/or CSF AFP level >1,000 ng/mL, associated with a negative prognostic impact on survival in the SIOP-96 trial (18). In our series, eight cases had CSF and/or serum AFP levels exceeding 1,000 ng/ml, with only one death related to disease. Due to the limited sample size, our series is unable to establish statistically-significant prognostic correlations.

Notably, the rarity of NGGCT, though linked with Down Syndrome in just one case in our series, warrants careful attention. A recent publication by Harris et al. revealed an increased risk of treatment-related adverse events and long-term neurocognitive sequelae in this patient group, necessitating alternative therapeutic approaches (24). Some innovative cases have been reported, such as ASCT and brentuximab-vedotin for those with CD30-positive embryonal carcinoma (25).

Diabetes insipidus is a common manifestation of germ cell tumors and an important risk factor for complications, especially during chemotherapy infusion using hyperhydration (26). Our two toxicity-related deaths emphasize the importance of education and management of endocrine complications, even in the long-term follow-up after completion of tumor treatment, to provide the best care for these patients (27, 28).

Our study’s limitations include the small patient cohort, underscoring the importance of collaborative efforts, particularly in countries with diverse populations like Brazil. Additionally, financial constraints prevented us from conducting biological studies, such as the recent discovery of 12p gain as a possible poor prognostic marker (29).

Despite these limitations, our study represents the largest series of NGGCT patients uniformly diagnosed and treated in an UMIC. It offers valuable insights into radiotherapy field strategies for localized disease and the ASCT approach for “slow responders”. Such feasible strategies in a collaborative setting contribute to global efforts aimed at improving outcomes for these patients.

## Data availability statement

The original contributions presented in the study are included in the article/**Supplementary Material**. Further inquiries can be directed to the corresponding author.

## Ethics statement

The studies involving humans were approved by Ethics Committee Federal University of Sao Paulo. The studies were conducted in accordance with the local legislation and institutional requirements. Written informed consent for participation in this study was provided by the participants’ legal guardians/next of kin.

## Author contributions

AC: Conceptualization, Data curation, Formal analysis, Funding acquisition, Investigation, Methodology, Project administration, Resources, Software, Supervision, Validation, Visualization, Writing – original draft, Writing – review & editing. ND: Conceptualization, Data curation, Formal analysis, Investigation, Methodology, Writing – original draft, Writing – review & editing. BM: Data curation, Writing – review & editing. SE: Data curation, Writing – review & editing. DA: Conceptualization, Data curation, Writing – review & editing. SC: Conceptualization, Data curation, Writing – review & editing. PD: Conceptualization, Data curation, Writing – review & editing. MA: Conceptualization, Data curation, Writing – review & editing. JN: Conceptualization, Data curation, Writing – review & editing. MCo: Conceptualization, Data curation, Writing – review & editing. FS: Conceptualization, Data curation, Writing – review & editing. SA: Conceptualization, Data curation, Writing – review & editing. MF: Conceptualization, Data curation, Writing – review & editing. MCh: Conceptualization, Data curation, Writing – review & editing. NS: Conceptualization, Data curation, Investigation, Writing – review & editing. JF: Conceptualization, Investigation, Writing – review & editing.
