# Supplementary material for: Intracranial non-germinomatous germ cell tumors in children and adolescents: how can the experience from an uppermiddle-income country contribute to the worldwide effort to improve outcomes?
Source: Front Oncol. 2024 Mar 4;14:1308128. doi: 10.3389/fonc.2024.1308128 (PMC10947194; doi:10.3389/fonc.2024.1308128)
Supplement: Supplementary file 1 [file DataSheet_1.pdf]

ELIGIBILITY CRITERIA

• Consent and/or informed consent obtained from legal guardian or patient. Patients' legal guardians consented to treatment and the patients themselves assented (children 6 years of age or older) or consented (18 years of age or older) when age was appropriate.

• Patients with newly-diagnosed intracranial non-germinoma regardless of age, without irradiation or previous chemotherapy (except corticosteroids), with biopsy proven and/or serum/CSF tumor marker elevations according to recommended values.

• Human chorionic gonadotropin – beta (HCGß) and alpha-fetoprotein (AFP) levels in both lumbar CSF and serum should be determined in the perioperative period.

• CSF collection for the determination of leptomeningeal dissemination by cytology should be performed pre, intraoperatively or 14 days after surgery, by lumbar puncture preferably, and reported at the time of patient registration.

• Cranial and total spinal MRI with and without contrast must be obtained in the preoperative period and 48 hours perioperatively or after 14 days of surgery, and the results known at the time of registration.

• Documented organ function: Patients must have adequate liver function (bilirubin less than 2.0mg/dL and transaminases (AST and ALT) less than 5 times the upper limit of normal. •Patients with indirect hyperbilirubinemia due to Gilbert's Syndrome will be eligible for entry into the study despite increased serum bilirubin •Renal function with normal urea and creatine and/or creatinine clearance, if possible, greater than 60 ml/min/1.73m2, •Cardiac function within normal limits, if possible, documented by echocardiogram, achieving ejection greater than 30%.

INELIGIBILITY CRITERIA

• Patients were excluded with no consent and/or informed consent were obtained.

• Patients with previous treatment (chemotherapy or irradiation).

• Patients without biopsy and/or tumor markers according to the recommended values of the protocol.

• Patients without cranio and spinal MRI and CSF obtained out of the recommended time in the protocol.

• Any patient with unacceptable morbidity from organs other than the CNS will be excluded from the study.

DIAGNOSIS

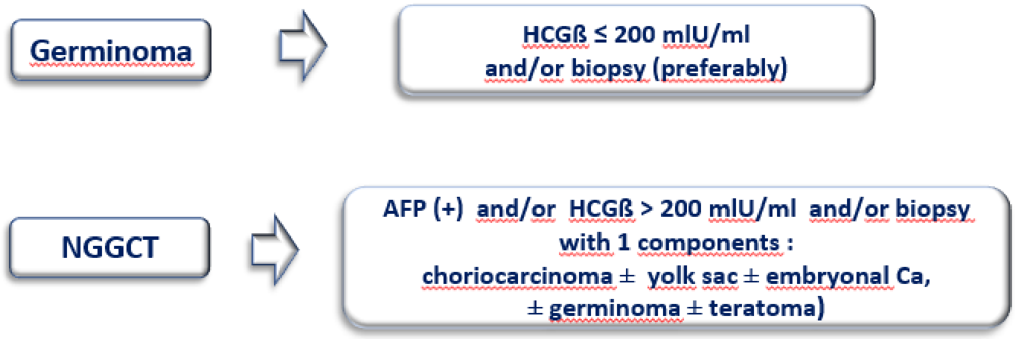

SCHEMA AND TREATMENT PLAN

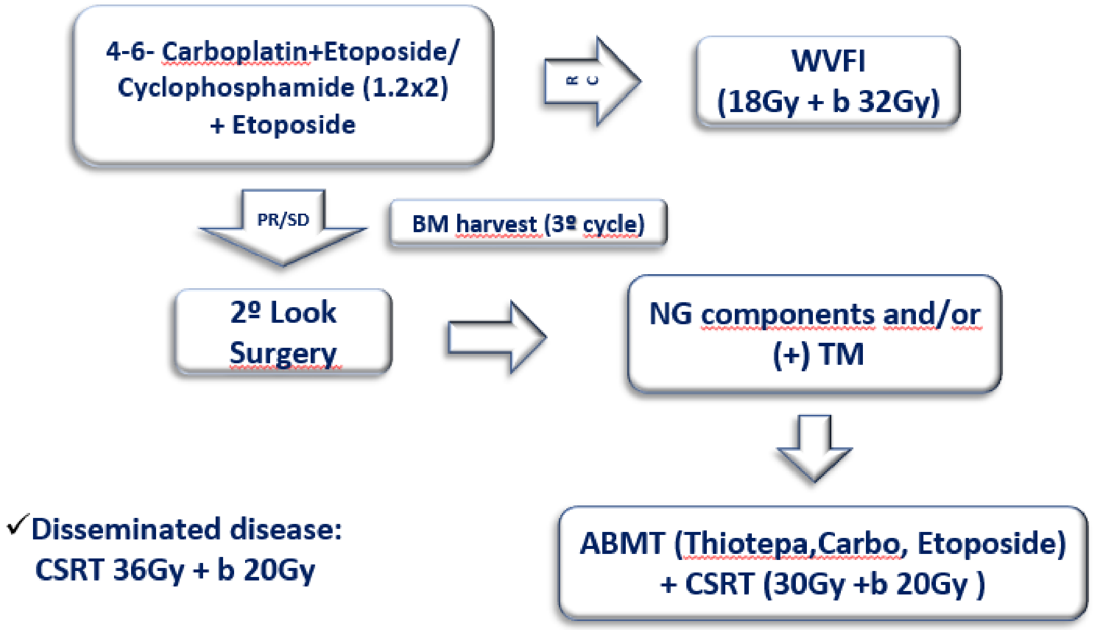

RULES FOR DOSE MODIFICATION

• Prior to each cycle, the absolute neutrophil count was required to be >500/mm^3, platelet count >100.000/mm^3, creatinine level < 1.5 mg/dL, transaminases < 5x the institutional normal level and bilirubin < 2.0mg/dL. Therapy was delayed if the patient did not meet these criteria, but episodes of fever and slow recovery neutropenia would not require dose reduction in subsequent cycles.

• In case of absolute neutrophil < 500/mm^3, granulocyte colony-stimulating factor (G-CSF) should be used (5mcg/kg/day, subcutaneous) until post-nadir white cell count be greater than 10,000/mm^3.

MEASUREMENT OF TREATMENT EFFECT – response criteria and methods

• Craniospinal MRI with and without gadolinium contrast and serum and lumbar CSF tumor marker assessments were performed after the second and fourth 4th chemotherapy cycles, approx. 6 weeks following completion of irradiation, every four months in the first three years after treatment, every six months until the fifth year and annually thereafter.

• Tumor measurements were assessed by a certified neuro-radiologists at each of the participating institutions and response was graded using the revised RECIST criteria (response evaluation criteria in solid tumors): Complete response (CR) was defined as no radiological evidence of tumor and normalization of both serum and lumbar CSF tumor markers; Partial response (PR) as 50% reduction in the product of the two greatest tumor diameters on imaging and reduction of previously elevated HCGB levels in both serum and lumbar CSF; Minor Response (MR) as 25-50% reduction on imaging and some reduction of previously elevated serum and lumbar CSF HCGß; Stable disease (SD) as <25% decrease in imaging size and Progressive disease (PD), 25% increase in the tumor size or increasing elevations of either HCGB or AFP in either serum or lumbar CSF.

DEFINITIONS OF SURVIVAL AND METHODS

• The Nonparametric event-free (EFS) and overall survival (OS) curves were calculated using the product-limit (Kaplan-Meier) estimates.

• EFS was defined as the time to disease progression, disease relapse, occurrence of a second neoplasm or death from any cause measured from the time of study enrollment.

• OS was defined as the time elapsed from diagnosis to the time of death due to all causes or the last follow-up visit.

• All statistical analyses were performed using IBM SPSS software for windows (version 29.0)

REASONS FOR EARLY CESSATION OF TRIAL THERAPY

• Progressive disease;

STATISTICAL SECTION

• A multicenter prospective trial of patients diagnosed with intracranial non-germinoma treated at IOP/GRAACC/Federal University of São Paulo (UNIFESP), Hospital do Amor de Barretos, Hospital Santa Marcelina/TUCCA, São Paulo State, Brazil, was performed between 2013 and 2021. Data were analyzed in December 2022.

• Patient characteristics were described using the absolute and relative frequencies for the qualitative variables and average, median, minimum and maximum values for the quantitative variables.

• The primary objectives of this study were to determine the event-free (EFS) and overall survival (OS) at 2- and 5-years of follow-up from diagnosis for patients with intracranial non-germinoma, to assess the impact on survival in reducing the dose of radiotherapy and examine the impact of ACST on the survival of NGGCT patients identified as 'slow responders'.

• The secondary objectives were to Implement second-look surgery for patients without radiological complete response (CR) following induction chemotherapy and observe its impact on survival.

• The Nonparametric event-free (EFS) and overall survival (OS) curves were calculated using the product-limit (Kaplan-Meier) estimates. EFS was defined as the time to disease progression, disease relapse, occurrence of a second neoplasm or death from any cause measured from the time of study enrollment. OS was defined as the time elapsed from diagnosis to the time of death due to all causes or the last follow-up visit.

• All statistical analyses were performed using IBM SPSS software for windows (version 29.0)
